# On Relationships between Gas-Phase Chemistry and Reactive Ion Etching Kinetics for Silicon-Based Thin Films (SiC, SiO_2_ and Si_x_N_y_) in Multi-Component Fluorocarbon Gas Mixtures

**DOI:** 10.3390/ma14061432

**Published:** 2021-03-15

**Authors:** Alexander Efremov, Byung Jun Lee, Kwang-Ho Kwon

**Affiliations:** 1 Department of Electronic Devices and Materials Technology, State University of Chemistry and Technology, 7 Sheremetevsky av., 153000 Ivanovo, Russia; amefremov@yandex.ru; 2 Department of Control and Instrumentation Engineering, Korea University, 2511 Sejong-ro, Sejong 30019, Korea; byung_jun@korea.ac.kr

**Keywords:** fluorocarbon gas plasma, etching kinetics, etching mechanism, plasma parameters, active species, polymerization, ion-assisted chemical reaction, effective reaction probability, etching yield

## Abstract

This work summarizes the results of our previous studies related to investigations of reactive ion etching kinetics and mechanisms for widely used silicon-based materials (SiC, SiO_2_, and Si_x_N_y_) as well as for the silicon itself in multi-component fluorocarbon gas mixtures. The main subjects were the three-component systems composed either by one fluorocarbon component (CF_4_, C_4_F_8_, CHF_3_) with Ar and O_2_ or by two fluorocarbon components with one additive gas. The investigation scheme included plasma diagnostics by Langmuir probes and model-based analysis of plasma chemistry and heterogeneous reaction kinetics. The combination of these methods allowed one (a) to figure out key processes which determine the steady-state plasma parameters and densities of active species; (b) to understand relationships between processing conditions and basic heterogeneous process kinetics; (c) to analyze etching mechanisms in terms of process-condition-dependent effective reaction probability and etching yield; and (d) to suggest the set gas-phase-related parameters (fluxes and flux-to-flux ratios) to control the thickness of the fluorocarbon polymer film and the change in the etching/polymerization balance. It was shown that non-monotonic etching rates as functions of gas mixing ratios may result from monotonic but opposite changes in F atoms flux and effective reaction probability. The latter depends either on the fluorocarbon film thickness (in high-polymerizing and oxygen-less gas systems) or on heterogeneous processes with a participation of O atoms (in oxygen-containing plasmas). It was suggested that an increase in O_2_ fraction in a feed gas may suppress the effective reaction probability through decreasing amounts of free adsorption sites and oxidation of surface atoms.

## 1. Introduction

In our days, silicon-based electronics still keep the dominant position in the worldwide production of various integrated circuits and discrete electronic devices. The chemical base of such devices is represented by the silicon itself, which is normally plays the role of the substrate material, as well as by three main silicon-based substances, such as SiC, SiO_2_ and Si_3_N_4_. In particular, silicon carbide is a very promising semiconductor featuring high thermal conductivity, high breakdown voltage and wide bandgap [1]. Accordingly, it has found numerous applications in high-power, high-frequency electronics (e.g., thyristors, static induction transistors, Schottky diodes and field-effect transistors) and/or in devices working at high temperatures [1,2,3]. Silicon dioxide and silicon nitride are a couple of widely used dielectric materials that appear as functional layers in various device structures, spacer dielectrics, passivating coatings and hard masks with high stability in respect to aggressive etchant environments [4,5]. In addition, the same applications are also for non-stoichiometric SiO_x_N_y_ films which also exhibit a dielectric nature and are characterized by both low density of surface states and high dielectric permittivity [6]. It was also found that SiO_x_N_y_ is a very attractive material for optical devices due to having quite low optical loss (less than 0.2 dB/cm at 1550 nm [7,8]) and a wide range of refractive index (between 1.45 for SiO_2_ and 2.0 for Si_3_N_4_) which may be adjusted through a change in the O/N ratio [8].

Obviously, since most real devices have complicated multi-layer structures, the corresponding device fabrication process needs the precision patterning of both the silicon substrate and preliminary deposited SiC, SiO, Si_3_N_4_ and/or SiO_x_N_y_ layers. Recently, strong requirements to devise dimension and performance pre-determine the use of “dry” patterning techniques, namely the reactive ion etching (RIE) method. The main feature of RIE is the simultaneous action of two parallel etching mechanisms, such as physical sputtering and ion-assisted chemical reaction [9,10]. In such a situation, an appropriate choice of working gas may allow flexible adjustment of the output process characteristics—etching rate, etching profile, selectivity in respect to mask and/or under-layer material, etching residues, surface roughness, etc. As for working gases, the leading position belongs to the fluorocarbon family with a general formula of C_x_H_y_F_z_ [9,10,11,12,13,14]. Inside the fluorocarbon gas family, CF_4_ exhibits the highest z/x ratio and, thus, is characterized by the domination of etching over surface polymerization for typical RIE conditions [9,13]. At the same time, fluorocarbon gases with z/x < 3 (for example, C_4_F_6_, C_4_F_8_, CHF_3_ and CH_2_F_2_) exhibit a high polymerization ability that results in the deposition of a fluorocarbon polymer film on the treated surface. Though the last phenomenon lowers absolute etching rates and causes etching residues, it positively influences the etching anisotropy for silicon (due to the passivation of side walls) as well as allowing one to obtain noticeably higher etching selectivity for couples of SiO_2_/Si and SiO_2_/Si_3_N_4_ (due to different thicknesses of polymer films on oxygen-free and oxygen-containing surfaces) [9,11]. Therefore, the adjustment of polymerizing ability through the proper selection of fluorocarbon gases, additive components and processing conditions provides an effective tool for the development of advanced RIE processes for silicon-based materials.

Until now, there have been many experimental studies (for example, Refs. [15,16,17,18,19,20,21,22,23] as well as earlier ones included in monographs [11,12,13,14]) that reported about RIE kinetics and mechanisms for silicon-based materials in various fluorocarbon gas plasmas. The most important findings of these works in respect to both etching and polymerization effects may be formulated as follows:

- The main contribution to the chemical etching pathway for all above-mentioned materials under typical RIE conditions (*p* < 50 mTorr, ion bombardment energy ~200–400 eV) belongs to F atoms. The role of CF_x_ (x = 1–3) radicals as etchant species is almost negligible; it works only through the ion-induced defluorination of deposited fluorocarbon polymer film [9,16].
- The steady-state thickness of polymer film, $h_{pol}$, under the same processing conditions normally decreases in the sequence of Si–Si_3_N_4_–SiO_2_ [15,16,17], as follows from an opposite order of corresponding etching rates [16,17]. The lowest $h_{pol,\;SiO_{2}}$ value is due to the etching of polymer by oxygen atoms on the film/SiO_2_ interface [9,17,18,19,20], while the condition $h_{pol,\;Si}$ > $h_{pol,\;Si_{3}N_{4}}$ is provided by the higher sticking probability of polymerizing radicals to the Si surface [16]. However, this rule may not work for all gas systems and processing conditions. As an example, Ref. [16] reports on the highest etching rate for Si_3_N_4_ in CHF_3_ plasma at ion energies above 150 eV.
- Both etching and polymerization kinetics may be effectively adjusted by mixing fluorocarbon gas with Ar and/or O_2_ [9,17,20,21,22,23]. Corresponding mechanisms work through changes in (a) the formation/decay balance for F atoms and polymerizing radicals in a gas phase; and (b) the physical and chemical decomposition rates of the fluorocarbon polymer film.
- The chemical interaction of F atoms with Si has no threshold energy and occurs spontaneously with the formation of highly volatile SiF_4_ at typical process temperatures [12,13,14]. That is why the “pure” Si + F reaction kinetics exhibits weak sensitivity to ion bombardment with energies below ~100 eV [11,12] (in fact, to the ion-stimulated desorption of reaction products) as well as being characterized by the exponent-like dependence of its etching rate on surface temperature [9]. At the same time, the RIE kinetics of silicon in high-polymerizing plasmas exhibits sufficient sensitivity to both ion flux and energy, in addition to the F atom density. The reasons are (a) the contribution of the sputter etching pathway; and (b) the change in $h_{pol}$ that influences the Si + F reaction probability through the access of F atoms to the etched surface [15,16,24].
- The chemical interaction of F atoms with SiC also has a threshold-less nature, since the strength of the Si–C bond of ~447 kJ/mol is lower compared with both Si–F (~552 kJ/mol [25]) and C–F (~514 kJ/mol [25]). Depending on the combination of processing conditions (type of fluorocarbon gas, additive components and ion bombardment energy), the SiC etching kinetics may correspond to either the neutral-flux-limited or the ion-flux-limited etching regime. Evidence of the first one is illustrated by the non-monotonic SiC etching rate vs. O_2_ fraction in the CF_4_ + O_2_ plasma [26,27] that corresponds to the change in F atom density [28,29,30]. Accordingly, the possibility of the ion-driven etching regime is indicated by the decreasing SiC etching rate vs. gas pressure [31]. Similarly to other materials, the SiC etching process in high-polymerizing fluorocarbons is affected by the deposition of polymer film [27,31].
- The chemical interaction of F atoms with Si_3_N_4_ can also follow a spontaneous mechanism (as the strength of the Si–N bond of ~470 kJ/mol [25] is smaller compared with that for Si–F) but shows much higher sensitivity to the ion bombardment intensity compared with Si [17]. This is due to the ion-induced destruction of Si–N bonds that creates energetically favorable adsorption sites for F atoms. Under typical RIE conditions, the Si_3_N_4_ etching process exhibits the features of a neutral-flux-limited regime controlled by F atom flux. The last conclusion follows from (a) an increase in Si_3_N_4_ etching with increasing gas pressure and input power [17,32] and (b) the non-monotonic (with a maximum at 30–40% O_2_) Si_3_N_4_ etching rate in the CF_4_ + O_2_ plasma [18,33,34] that corresponds to the similar non-monotonic behavior of F atom density [28,29,30].
- The chemical interaction of F atoms with SiO_2_ cannot occur spontaneously because of the sufficient energy threshold (as the Si-F bond energy is lower than the Si–O one, ~799 kJ/mol [25]). That is why the dry etching of SiO_2_ requires mandatory ion bombardment (to produce adsorption sites for F atoms through Si–O bonds breaking as well as to desorb low volatile non-saturated SiF_x_ compounds [9]) and is controlled by ion flux at ion energies below ~200 eV. At the same time, higher ion energies are generally enough to provide domination of the chemical etching pathway controlled by F atom flux [10,14]. The last feature is confirmed by the non-monotonic (with a maximum at 30–40% O_2_) SiO_2_ etching rate in the CF_4_ + O_2_ plasma [21,23], similar to those obtained for Si [9,12] and Si_3_N_4_ [18,33,34].

Analysis of the above data allows one to formulate at least two serious problems which require additional justifications and research efforts. Firstly, a significant part of published works have a mostly experimental nature and, thus, discuss both etching and polymerization effects without data on plasma parameters and plasma chemistry. As such, even the very detailed and accurate studies (for example, Refs. [15,16,17,18,19,20,21,22,23]) say nothing about mechanisms which transfer the change in operating conditions to heterogeneous process kinetics. Obviously, the unknown relationships between gas-phase and heterogeneous chemistries limit the significance of corresponding results (as the latter are surely valid only for a given combination of processing conditions) and cause uncertainties in interpretations of etching mechanisms. Secondly, insufficient attention was paid to the effect of gas mixing ratios in two- or three-component gas mixtures which combine either one fluorocarbon component with two additives or two fluorocarbons with one additive gas. At the same time, some published works [35,36] clearly demonstrated that the change in gas mixing ratios in two- and, especially, in three-component gas systems may be an actual tool to control both gas-phase plasma parameters and output etching characteristics such as etching rate, etching anisotropy, etching selectivity in respect to both mask and under-layer materials as well as etching uniformity and surface roughness. Obviously, the absence of systematic studies in this direction does not help to understand the features and applicability of a given gas system for the purpose of a given etching process.

In recent years, we performed a series of research works dealing with effects of gas mixing ratios in two- and three-component fluorocarbon gas mixtures [36,37,38,39,40,41,42,43]. In these works, etching experiments were combined with plasma diagnostics by Langmuir probes and 0-dimensional plasma modeling. Such an approach allows one (a) to understand how the gas mixing ratio influences electron- and ion-related plasma parameters at constant processing conditions (gas pressure, input power and bias power); (b) to figure out key plasma chemical processes determining the steady-state densities of F atoms and polymerizing radicals; (c) to suggest a set of gas-phase-related variables (in the form of species fluxes and flux-to-flux ratios) in order to trace the polymer deposition/decomposition kinetics; and d) to match changes of RIE process characteristics with those in gas-phase and heterogeneous reaction kinetics. The main idea of the present work was to summarize and re-discuss, on a comparative scale, our results concerning CF_4_ (z/x = 4)–, CHF_3_ (z/x = 3)– and C_4_F_8_ (z/x = 2)-based gas mixtures. These gases were chosen because of the continuous decrease in the z/x ratio in the sequence of CF_4_–CHF_3_–C_4_F_8_ that pre-determines mandatory differences in the densities of polymerizing radicals and fluorine atoms. Accordingly, the comparison of corresponding results may provide the ability for better understanding the features of reactive ion etching processes in low- and high-polymerizing gas systems. The subjects of our interest were their binary mixtures with Ar (as the simplest gas systems where the additive gas influences both gas-phase and heterogeneous process kinetics only through physical effects), ternary mixtures which combine the fluorocarbon component with Ar and O_2_ (as gas systems where reactions involving O and O(^1^D) species influence the densities of active species and polymer decomposition rates [9,10,11,12]) as well as ternary mixtures with two fluorocarbon components. In the last case, it was suggested that mixing two fluorocarbons with different z/x ratios allows wide-range adjustment of both etching and polymerization kinetics in oxygen-less gas systems. Accordingly, the component mixing ratio always played the role of the main variable parameter, and the most attention was paid to the following issues:

- To understand how the chemical nature of the fluorocarbon component and corresponding z/x value influences electron- and ion-related plasma parameters (electron temperature, plasma density, ion flux and energy) that determine both electron impact kinetics and ion–surface interaction efficiency;
- To figure out differences in steady-state densities of F atoms and polymerizing radicals under the same processing conditions in light of their formation/decay kinetics as well as to evaluate the ability of additive gases (rather, the impact of their mixing ratios) to adjust gas-phase compositions in corresponding gas systems;
- To compare RIE performances for both non-oxygenated and oxygenated gas systems with respect to various silicon-based materials in terms of etching rates and etching selectivity as well as to suggest corresponding etching mechanisms and limiting stages through correlations between fluxes of active species and obtained etching kinetics.

## 2. Materials and Methods

### 2.1. Experimental Setup and Procedures

**Figure 1:**
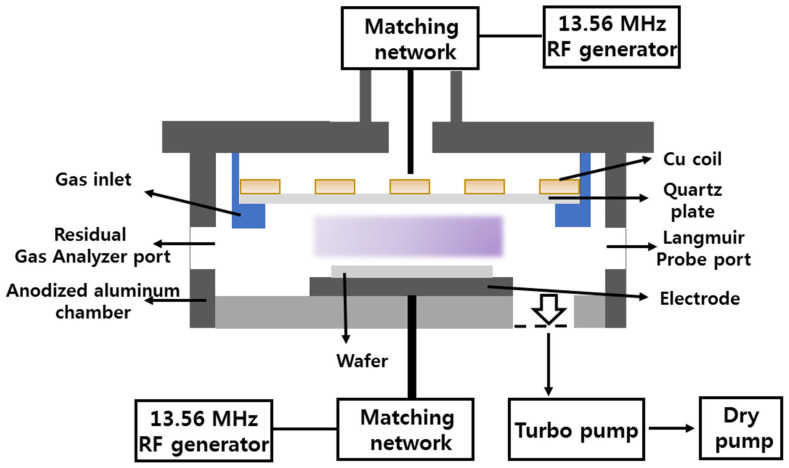
Schematic diagram of inductively coupled plasma (ICP) reactor with arrangement.

Experiments were carried out in a planar inductively coupled plasma (ICP) reactor (Figure 1), the same as that used in our previous works [36,37,38]. The reactor chamber was made from anodized aluminum and had a cylindrical (r = 16 cm, l = 13 cm) shape. Plasma was produced using the rf (radio frequency 13.56 MHz) power supply which was connected to the flat 5-turn copper antenna through the matching network. The antenna was located on the top side of the chamber and was separated from the vacuum part by a quartz window. Another 13.56-MHz rf power supply biased the bottom electrode (the substrate holder) in order to set the ion bombardment energy through the negative dc bias voltage ($-U_{dc}$). The last parameter was measured using a high-voltage probe (AMN-CTR, Youngsin-RF Co.,Ltd, Seoul, Korea). The bottom electrode was equipped with a water flow cooling system that allows one to maintain nearly constant temperatures ($T_{S}$) for the processing times τ ~ 5 min. The variable processing parameters were total gas flow rate (q = 40–60 sccm), gas pressure (p = 4–10 mTorr), input power (W = 600–900 W), bias power ($W_{dc}$ = 200–300 W) and component mixing ratios in the feed gas. The latter were set by adjusting partial flow rates of individual gases ($q_{i}$) under the condition of q = const. Accordingly, the composition of the input gas mixture was characterized by component fractions $y_{i}=q_{i}/q$.

Plasma diagnostics by the double Langmuir probe (DLP2000, Plasmart Inc., Deajeon, Korea) provided experimental data on electron- and ion-related plasma parameters such as electron temperature ($T_{e}$) and ion current density ($J_{+}$). The treatment of measured I–V curves was based on the double Langmuir probe theory in collision-less probe sheath approximation [44,45]. In order to minimize the distortion of both raw I–V curves and related data due to the contamination of probe tips by the fluorocarbon polymer, the latter were cleaned in 50% Ar + 50% O_2_ plasma before and after each measurement. From our previous works (for example, Refs. [35,36]), it can be understood that such a procedure provides adequate diagnostics results in high-polymerizing fluorocarbon plasmas.

Etching kinetics for Si, SiC, SiO_2_ and Si_3_N_4_ (or the non-stoichiometric SiN_x_) were studied using fragments of Si (100) wafers without or with a preliminary deposited layer of the corresponding material. SiC films with a thickness of ~150 nm were produced by rf magnetron sputtering of 99% SiC target in Ar plasma. The substrate surface was preliminarily cleaned from organic contaminations through consequent washing in trichloroethylene, acetone, methanol and deionized water. More details about the deposition method and regimes are available in Ref. [46]. SiO_2_ films with a thickness of ~500 nm were produced using the low-pressure chemical vapor deposition (LPCVD) technique. The precursor gases were SiH_4_ and O_2,_ and the substrate temperature was 425 °C. More details about the deposition method and regimes may be found in Ref. [40]. SiN_x_ films with a thickness of ~400 nm were produced by the plasma-enhanced chemical vapor deposition (PECVD) method. The precursor gases were SiH_4_, He, NH_3_ and N_2_. The deposition process was carried out in an rf (13.56 MHz) plasma source operated at 300 W and 800 mTorr. The substrate temperature was 150 °C. More details about the deposition method and regimes may be found in Ref. [40]. Ellipsometry measurements (EC-400 and M-2000V, J.A Woollam, Lincoln, NE, USA) showed an average refractive index of 2.026 ± 0.001 in the wavelength range of 370–990 nm. This value is quite close to that for stoichiometric Si_3_N_4_ (~2.0 [25]).

Etched samples with a size of ~2 cm × 2 cm were placed on the bottom electrode and centered in radial direction. The small sample surface area aimed to reduce the loading effect as well as to provide an etching regime reflecting actual heterogeneous kinetics. In preliminary experiments, no principal differences were found in both measured I–V curves and matched plasma parameters obtained with and without sample loading. In fact, this allows one to neglect the sensitivity of gas-phase plasma characteristics to etching products as well as to consider the gas phase to be the permanent source of active species. Etched depths (∆h) were measured using the surface profiler Alpha-Step 500 (Tencor, Milpitas, CA, USA) for the processing time τ = 1 min. In order to supply these measurements, we provided a partial surface masking using the photoresist AZ1512 with a thickness of ~1.5 µm. The quasi-linear shapes of $∆h=f\left(\tau \right)\;$ curves in all investigated gas systems pointed out the steady-state etching kinetics as well as allowing us to assume the very weak change in the sample temperature, at least within the given processing time. Otherwise, the continuously increasing sample temperature is expected to produce Arrhenius-like $∆h=f\left(\tau \right)$ curves instead of nearly linear ones (at least for Si due to its spontaneous reaction mechanism with F atoms). Based on the above data, etching rates were always calculated as R=∆h/τ, while we ignored sample temperature-related effects when analyzing changes in etching kinetics vs. gas mixing ratio at p, W = const.

### 2.2. Approaches for Analysis of Gas-Phase Chemistry

In order to obtain information on the kinetics and densities of plasma active species, we applied a simplified 0-dimensional (global) kinetic model [47,48]. A set of chemical reactions with corresponding rate coefficients was taken from our previous works that dealt with the modeling of CF_4_ + Ar/O_2_ [40,47,48,49,50], CHF_3_ + Ar/O_2_ [50,51,52,53] and C_4_F_8_ + Ar/O_2_ [36,39,43,48,54] plasmas. The basic model approaches were as follows:

- The electron energy distribution function (EEDF) has a nearly Maxwellian shape. The applicability of Maxwellian EEDFs to describe electron-impact kinetics in low-pressure CF_4_–, CHF_3_– and C_4_F_8_– based plasmas has been demonstrated in several works [28,55,56,57,58]. This allows one to obtain electron-impact rate coefficients as $k=AT_{e}{}^{B}\exp\left(-C/T_{e}\right)$ [55,56,58] based on measured $T_{e}$ values.
- The dependence of gas temperature, $T_{gas}$, on gas mixing ratios at p, W = const. may be neglected. The indirect proof for this suggestion is the nearly constant temperature of the external chamber wall obtained for different gas mixing ratios at fixed plasma on time. Since experimental data on $T_{gas}$ were not available in this study, we took 600–700 K as the typical value for close processing conditions, reactor type and geometry [47,48,49,50,51,52,53]. This allowed us to operate with constant rate coefficients for gas-phase atom molecular reactions, which were taken from the NIST(National Institute of Standards and Technology) Chemical Kinetics Database [59].
- The heterogeneous loss of atoms and radicals is described by first-order recombination kinetics with $k\approx \gamma \upsilon_{T}/\Lambda$ [36,37,38], where $\Lambda ={\left[{\left(2.405/r\right)}^{2}+{\left(\pi /l\right)}^{2}\right]}^{-1/2}\;$ is the diffusion length [9] and $\upsilon_{T}=\sqrt{8k_{B}T_{gas}/\pi m}$ is the thermal velocity for a particle with a mass of m. Recombination probabilities γ were taken from Refs. [55,56,58,60] where these were obtained experimentally or adjusted by a plasma modeling procedure. We also suggested that all recombination probabilities are independent of gas mixing ratios because of the stable chamber wall conditions. The reasons are (a) no re-deposition of reaction products due to the low sample size; and (b) the nearly constant wall temperature. The indirect proof for the last suggestion is the nearly constant external wall temperature, as was mentioned above.
- The electronegativity of low-pressure CF_4_–, CHF_3_– and C_4_F_8_– based plasmas, even with addition of O_2_, is low enough to assume $n_{e}\approx n_{+}$ [28,55,56,57,58], where $n_{e}$ is the electron density and $n_{+}$ is the total density of positive ions. The latter was obtained from measured $J_{+}$ without accounting for the influence of negative ions on the ion Bohm velocity:
  (1) $$n_{+}\approx \frac{J_{+}}{0.61e\sqrt{eT_{e}/m_{i}}}\;$$

The effective ion mass was evaluated according to Blank’s law as $m_{i}={\left(\sum y_{X^{+}{}_{i}}/m_{X^{+}{}_{i}}\right)}^{-1}$, where $y_{X^{+}{}_{i}}$ and $m_{X^{+}{}_{i}}$ are partial ion fractions and masses, respectively. For each type of positive ion, it was suggested that $y_{X^{+}}\sim k_{iz}y_{X}/\sqrt{1/m_{X^{+}}}$ [40], where $y_{X}$ is the fraction of corresponding neutral particles with the ionization rate coefficient of $k_{iz}$ [55,58].

The output model parameters were the volume-averaged steady-state densities of plasma active species and their fluxes to the etched surface. It should also be noted that the combination of the used modeling algorithm and kinetic scheme provides an adequate description of the plasma chemistry in the given gas systems [55,56,57,58].

### 2.3. Approaches for Analysis of Etching Kinetics

The basic features of reactive ion etching processes in fluorocarbon-based plasmas as well as related reaction mechanisms for Si, SiO_2_ and Si_3_N_4_ have been discussed in detail in Refs. [15,16,18,19,20,24,36,37,38,39,61,62]. When summarizing these findings, the following general approaches for the analysis of etching kinetics may be used:

- Under reactive ion etching conditions (i.e., when the ion bombardment energy exceeds the sputtering threshold for the target surface), the measured etching rate, R, may be represented in the form of two summands, $R_{phys}+R_{chem}$ [9,61]. These represent physical (sputtering by ion bombardment) and chemical (ion-assisted chemical reaction) etching pathways, respectively.
- The ion-assisted chemical reaction has the rate of $\gamma_{R}\Gamma_{F}$ [37,38], where $\gamma_{R}$ is the effective reaction probability which accounts for the net effect from all heterogeneous stages, and $\Gamma_{F}\approx n_{F}\upsilon_{T}/4$ is the fluorine atom flux. In general, the effective reaction probability $\gamma_{R}\approx s_{0}\left(1-\theta \right)$ [38,39] depends on surface temperature (through the sticking coefficient $s_{0}$) as well as on any factor influencing the fraction of free adsorption sites for F atoms $\left(1-\theta \right)$. In polymerizing plasmas, $\gamma_{R}$ decreases with increasing thickness of the fluorocarbon polymer film when the latter comes to be enough to provide $\Gamma_{F}{}^{\prime }/\Gamma_{F}$ << 1, where $\Gamma_{F}{}^{\prime }$ is the flux of F atoms on the polymer film/etched surface interface [39].
- The physical sputtering (as well as any other ion-related etching effect with a purely physical nature: breaking of chemical bonds between surface atoms, ion-stimulated desorption of reaction products) has the rate of $Y_{S}\Gamma_{+}$ [9,63], where $Y_{S}$ ~ $\sqrt{\;M_{i}\varepsilon_{i}}$ [35,36,37] is the sputtering yield, $\;M_{i}=\;m_{i}\;N_{A}$ is the effective ion molar mass, $\varepsilon_{i}=\left|-U_{f}-U_{dc}\right|$ is the ion bombardment energy, $-U_{f}\approx 0.5T_{e}\ln\left(m_{i}/2\pi m_{e}\right)$ is the floating potential and $\Gamma_{+}\approx J_{+}/e$ is the flux of positive ions. Accordingly, the relative change in the corresponding process rate may be traced by the parameter $\sqrt{M_{i}\varepsilon_{i}}\Gamma_{+}$ [37,38]. Obviously, in the case of $M_{i}$ const., one can simply use $\sqrt{\varepsilon_{i}}\Gamma_{+}$.
- The growth of the fluorocarbon polymer film is provided by the CF_x_ (x = 1, 2) radicals, and the polymerization ability increases in fluorine-poor plasmas [16,24]. Accordingly, the polymer deposition rate may be traced by the $\Gamma_{pol}/\Gamma_{F}$ ratio [35,36,37], where $\Gamma_{pol}$ is the total flux of polymerizing radicals ($\Gamma_{CF_{2}}+\Gamma_{CF}$ in CF_4_- and C_4_F_8_- based plasmas while $\Gamma_{CF_{2}}+\Gamma_{CF}\;+\Gamma_{CHF}$ in CHF_3_- based plasmas). Accordingly, parameters $\Gamma_{pol}/\sqrt{M_{i}\varepsilon_{i}}\Gamma_{+}\Gamma_{F}$ and $\Gamma_{pol}/\Gamma_{O}\Gamma_{F}$ characterize the change in the polymer film thickness due to physical (destruction by ion bombardment) and chemical (etching by O atoms) destruction pathways, respectively [35,36,40,48].

## 3. Results

### 3.1. Non-Oxygenated Gas Systems

**Figure 2:**
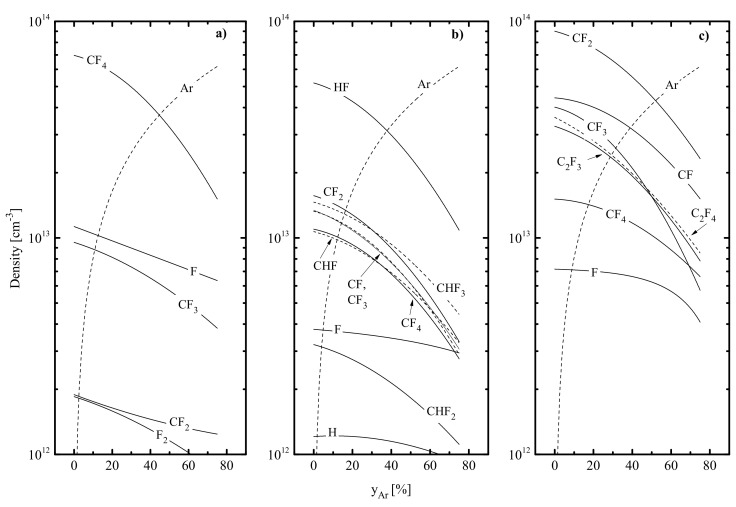
Densities of neutral species in CF_4_ + Ar (**a**), CHF_3_ + Ar (**b**) and C_4_F_8_ + Ar (**c**) plasmas at p = 6 mTorr, W = 700 W and $W_{dc}$ = 200 W

**Figure 3:**
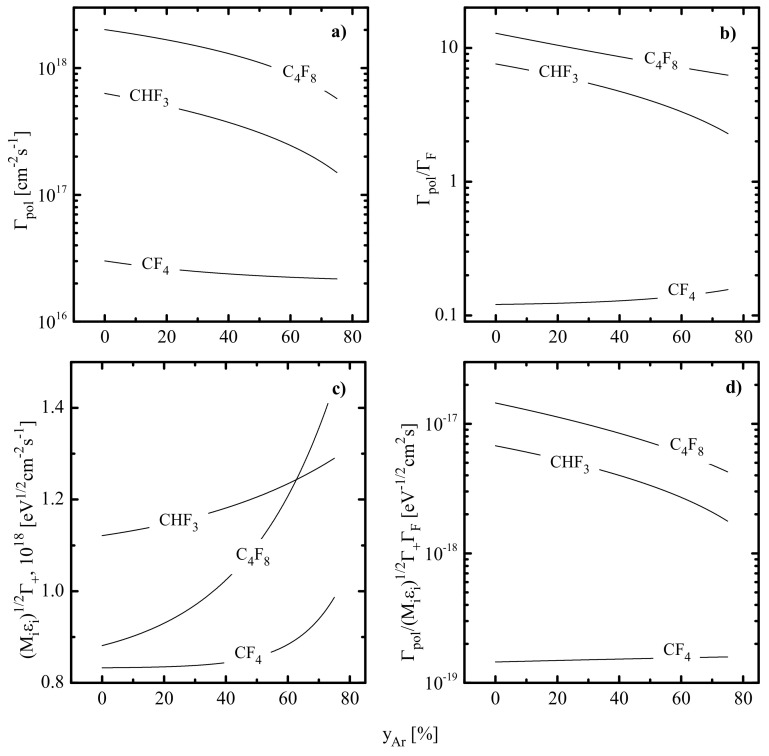
Fluxes and flux-to-flux ratios characterizing etching and polymerization kinetics in CF_4_ + Ar, CHF_3_ + Ar and C_4_F_8_ + Ar plasmas at p = 6 mTorr, W = 700 W and $W_{dc}$ = 200 W: (**a**) total flux of polymerizing radicals; (**b**) $\Gamma_{pol}/\Gamma_{F}$ ratio characterizing polymer deposition rate; (**c**) parameter $\sqrt{M_{i}\varepsilon_{i}}\Gamma_{+}$ characterizing polymer etching rate and (**d**) parameter $\Gamma_{pol}/\sqrt{M_{i}\varepsilon_{i}}\Gamma_{+}\Gamma_{F}$ characterizing the change in polymer film thickness due to the physical etching pathway.

The influence of input RIE process conditions (gas pressure and flow rate, input power and bias power) on electron- and ion-related plasma parameters in CF_4_, CHF_3_ and C_4_F_8_ gases has been studied in earlier works [55,56,57,58,64,65,66]. That is why, below, we will focus the attention only on their key properties which seem to be principal for understanding the features of CF_4_ + Ar, CHF_3_ + Ar and C_4_F_8_ + Ar plasmas under the condition of p, W = const. These are as follows:

- The electron temperature increases in the sequence of CF_4_–C_4_F_8_–CHF_3_, while the shapes of $T_{e}=f\left(y_{Ar}\right)$ curves in corresponding gas mixtures are somewhat different (Table 1). The maximum value of $T_{e}$ in the CHF_3_ plasma results from low electron energy losses for the ionization of the dominant neutral component HF (Hydrogen Fluoride) compared with that for CF_4_ molecules in the CF_4_ plasma and CF_2_ radicals in the C_4_F_8_ plasma (Figure 2). Such a conclusion directly follows from the comparison of rate coefficients (k) and threshold energies (ε) for R1: HF + e → HF^+^ + 2e ($\varepsilon_{1}$ = 16.1 eV, $k_{1}$ = 4.6 × 10^−10^ cm^3^/s at $T_{e}$ = 3 eV); R2: CF_4_ + e → CF3^+^ + F + 2e ($\varepsilon_{2}$ = 15.9 eV, $k_{2}$ = 1.6 × 10^−9^ cm^3^/s at $T_{e}$ = 3 eV) and R3: CF_2_ + e → CF_2_^+^ + 2e ($\varepsilon_{3}$ = 10.0 eV, $k_{3}$ = 2.1 × 10^−9^ cm^3^/s at $T_{e}$ = 3 eV). The condition $T_{e}$ (CF_4_) < $T_{e}$ (C_4_F_8_) is connected with higher electron energy losses for vibrational and electronic excitations for CF_4_ compared with CF_2_. An increase in $y_{Ar}$ in the CF_4_ + Ar gas mixture causes a nearly proportional decrease in electron energy losses for vibrational and electronic excitations of CF_x_ species as well as leads to the opposite change in energy losses for ionization due to R4: Ar + e → Ar^+^ + 2e ($\varepsilon_{4}$ = 15.6 eV, $k_{4}$ = 3.0 × 10^−9^ cm^3^/s at $T_{e}$ = 5 eV). Accordingly, the change in CF_4_/Ar mixing ratio at p, W = const. results in $T_{e}$ ≈ const. At the same time, an increase in $y_{Ar}$ in CHF_3_ + Ar and C_4_F_8_ + Ar plasmas is characterized by much a stronger increase in electron energy losses for ionization (due to $\varepsilon_{1}k_{1}$ << $\varepsilon_{2}k_{2}$ and $\varepsilon_{3}k_{3}$) that produces a decrease in $T_{e}$ (Table 1).
The plasma density increases in the sequence of C_4_F_8_–CF_4_–CHF_3_ (Table 1) due to the same order of total ionization frequencies, as follows from $k_{1}$ < $k_{2}$ < $k_{3}$. Similar changes in both $n_{+}$ and $n_{e}$ vs. Ar fraction in the feed gas in all three gas systems result from an increase in total ionization frequencies toward Ar-rich plasmas (2.7 × 10^4^–6.5 × 10^4^ s^−1^ for CF_4_ + Ar; 1.2 × 10^5^–1.7 × 10^5^ s^−1^ for CHF_3_ + Ar and 9.5 × 10^4^–2.1 × 10^5^ s^−1^ for C_4_F_8_ + Ar at 0–75% Ar). Mechanisms providing such a phenomenon are the condition $k_{4}$ > $k_{1}$, $k_{2}$ and $k_{3}$ as well as the contribution of R5: Ar^m^ + e → Ar+ + 2e, where Ar^m^ = Ar (^3^P_0,1,2_) are the metastable Ar atoms. Accordingly, it is analogous for ion current densities $J_{+}$ (0.95–1.63 mA/cm^2^ for CF_4_ + Ar; 1.55–2.20 mA/cm^2^ for CHF_3_ + Ar and 1.05–2.36 mA/cm^2^ for C_4_F_8_ + Ar at 0–75% Ar) that determine ion fluxes to the etched surface $\Gamma_{+}$. The local disturbance of this rule for CF_4_ + Ar and C_4_F_8_ + Ar plasmas at $y_{Ar}$ < 30% ($J_{+,C_{4}F_{8}}$ > $J_{+,CF_{4}}$ in spite of $n_{+,C_{4}F_{8}\;}$ < $n_{+,CF_{4}\;}$) is connected with differences in masses and transport coefficients for dominant positive ions.
The negative dc bias at $W_{dc}$ = const. exhibits a growth in the sequence of CHF_3_–CF_4_–C_4_F_8_ as well as decreases with increasing Ar fraction in a feed gas (Table 1). This reasonably causes the same order and behaviors of ion bombardment energies $\varepsilon_{i}$ (285–211 eV for CF_4_ + Ar, 261–208 eV for CHF_3_ + Ar and 307–221 eV for C_4_F_8_ + Ar at 0–75% Ar). Quite close values of $\sqrt{M_{i}\varepsilon_{i}}$ in all three gas systems allow one to conclude that corresponding differences in the efficiency of ion bombardment are mostly related to those in ion fluxes. From Figure 3, it can be understood that highest and lowest values for the parameter $\sqrt{M_{i}\varepsilon_{i}}\Gamma_{+}$ under the condition of $y_{Ar}$ < 60% are in CHF_3_ + Ar and CF_4_ + Ar plasmas, respectively. At the same time, an increase in Ar fraction above 60% changes the situation due to the rapid increase in $\Gamma_{+}$ in the C_4_F_8_ + Ar plasma.

Figure 2 represents the model-predicted data on the steady-state densities of neutral species in CF_4_ + Ar, CHF_3_ + Ar and C_4_F_8_ + Ar plasmas. From Figure 2a, one can see that the dominant neutral components in pure CF_4_ plasma are original CF_4_ molecules and F atoms [51,53,55]. The decreasing densities of fluorocarbon radicals in the sequence of CF_3_–CF_2_–CF [55,66] are due to both the step-by-step formation pathway in electron-impact processes R6: CF_x_ + e → CF_x−1_ + F + e as well as higher heterogeneous loss probabilities for less saturated species. The main source of fluorine atoms (~80% from the total formation rate) is represented by R6 for x = 3, 4 together with the dissociative ionization R7: CF_4_ + e → CF_3_^+^ + F + 2e. Accordingly, the decay of atomic species is provided by heterogeneous processes R8: F + F(s) → F_2_ and R9: F + CF_x_(s) → CF_x+1_, where index “(s)” points out the adsorbed state of corresponding particles. According to Figure 2b, the dominant neutral components in pure CHF_3_ plasma are HF, CHF_3_ and CF_x_ (x = 1–3). The high density of HF (which is in good agreement with Refs. [65,66,67]) is provided by two mechanisms: a) the direct formation of these species in R10: CHF_3_ + e → HF + CF_2_ + e; and 2) the high efficiency of gas-phase reactions R11: CHF_x_ + F → CF_x_ + HF ($k_{11}$ ~ 3.0 × 10^−11^ cm^3^/s for x = 1, 2), R12: CHF_x_ + H → CHF_x−1_ + HF ($k_{12}$ ~ 3.0 × 10^−10^ cm^3^/s for x = 1, 2) and R13: CF_x_ + H → CF_x−1_ + HF ($k_{13}$ ~ 8 × 10^−11^ cm^3^/s for x = 3 and ~ 4 × 10^−11^ cm^3^/s for x = 2). That is why the main formation pathways for F atoms are R14: HF + e → H + F + e and R6 for x = 2, 3. It is important to note, also, that the contribution of R11 to the total decay rate for F atoms exceeds those of R8 and R9. As follows from Figure 2c, the gas phase of pure C_4_F_8_ plasma is composed by fluorocarbon components CF_x_ (x = 1, 2, 3) and C_2_F_x_ (x = 3, 4) [57,58,60]. These particles appear as the first-step dissociation products of original C_4_F_8_ molecules in R15: C_4_F_8_ + e → 2C_2_F_4_ + e and R16: C_4_F_8_ + e → C_3_F_6_ + CF_2_ + e as well as resulting from further decomposition of the corresponding reaction products through R6 for x = 2, R17: C_3_F_6_ + e → C_2_F_4_ + CF_2_ + e, R18: C_2_F_4_ + e → 2CF_2_ + e and R19: C_2_F_4_ + e → C_2_F_3_ + F + 2e. The main source of F atoms is given by R6 for x = 1, 2, 3 while their decay is noticeably contributed by R20: C_2_F_4_ + F → CF_2_ + CF_3_ ($k_{18}$ ~ 4 × 10^−11^ cm^3^/c).

When analyzing data of Figure 2, one can formulate some principal conclusions concerning both the effect of Ar on gas-phase plasma characteristics and the possible reflections of corresponding phenomena on etching process kinetics. These are as follows:

- The total density of polymerizing radicals $n_{pol}$ ($n_{CF}+n_{CF_{2}}$ for CF_4_ + Ar and C_4_F_8_ + Ar plasmas while $n_{CF}+n_{CF_{2}}+n_{CHF}$ for the CHF_3_ + Ar plasma) increases in the sequence of CF_4_–CHF_3_–C_4_F_8_, and the gap between first and last gas systems reaches two orders of magnitude. Such a phenomenon is because the C_4_F_8_+Ar plasma provides the maximum production rate of CF_2_ radicals by R16, R18 and R20. An increase in $y_{Ar}$ causes a nearly proportional decrease in $n_{pol}$ in CHF_3_ + Ar (by ~4.2 times at 0–75% Ar) and C_4_F_8_ + Ar (by ~3.8 times at 0–75% Ar) plasmas but produces a much slower effect in the case of the CF_4_ + Ar gas system (by ~1.4 times at 0–75% Ar). The reason is the increasing dissociation frequencies for multi-atomic fluorocarbon species (16.2–34.4 s^−1^ for CF_4_ in R6 and R7, 28.8–51.0 s^−1^ for CF_3_ in R6 and 40.2–74.1 s^−1^ for CF in R6 at 0–75% Ar) due to an increase in both electron temperature and electron density. Accordingly, similar behaviors were also obtained for the total flux of polymerizing radicals to the etched surface $\Gamma_{pol}$ (Figure 3a).
- The density of fluorine atoms $n_{F}$ decreases in the sequence of CF_4_–C_4_F_8_–CHF_3_, and the gap between first and last gas systems reaches around three times. The lowest $n_{F}$ value in the CHF_3_ plasma results from the (a) low F atom formation rate because $k_{14}$ < $k_{6}$ and (b) high F decay rate in R11 that exceeds the total effect from R8 and R9. The much smaller difference in $n_{F}$ between CF_4_ + Ar and C_4_F_8_ + Ar plasmas (that is in good agreement with Ref. [68]) is only because the last system provides slightly higher F atom decay frequencies due to the contribution of R20. It can be understood, also, that as the Ar fraction in a feed increases, the F atoms’ density in all three gas systems decreases slower compared with $\left(1-y_{Ar}\right)$. In the CF_4_ + Ar plasma ($n_{F}$ = 1.1 × 10^13^ cm^−3^–6.4 × 10^12^ cm^−3^, or by ~1.8 times at 0–75% Ar), the reason for this phenomenon is the same behavior of the F atom formation rate in R6 and R7 due to increasing process frequencies, as was mentioned above. Oppositely, CHF_3_ + Ar and C_4_F_8_ + Ar plasmas exhibit nearly constant frequencies for R6, R7 and R14 (due to opposite changes in electron temperature and electron density) and, thus, a nearly proportional decrease in F atom formation rate with increasing Ar fraction in the feed gas. As such, the slow change in $n_{F}$ in these gas systems (3.8 × 10^12^ cm^−3^–3.0 × 10^12^ cm^−3^, or by ~1.3 times in CHF_3_ + Ar and 7.1 × 10^12^ cm^−3^–4.1 × 10^12^ cm^−3^, or by ~1.7 times in C_4_F_8_ + Ar at 0–75% Ar) is not connected with electron impact kinetics but results from rapidly decreasing F atom decay frequencies in gas-phase reactions R11 and R20. Similar tendencies were also obtained for the flux of fluorine atoms to the etched surface $\Gamma_{F}$.

The comparison of $\Gamma_{pol}/\Gamma_{F}$ ratios plotted in Figure 3b allows one to suggest that the polymer deposition rate exhibits the maximum value in C_4_F_8_ + Ar plasma and reaches the minimum in CF_4_ + Ar plasma. Therefore, the same order is also followed for the steady-state thicknesses of polymer films, as follows from Figure 3d. Obviously, the last conclusion seems to be reasonable and is in principal agreement with differences in z/x ratio for original fluorocarbon molecules. The evident feature of the CF_4_ + Ar plasma is that it provides nearly constant values for $\Gamma_{pol}/\Gamma_{F}$ and $\Gamma_{pol}/\sqrt{M_{i}\varepsilon_{i}}\Gamma_{+}\Gamma_{F}$ under the condition of $y_{Ar}$ = 0–75%. In fact, this means a weak effect of CF_4_/Ar mixing ratio on both surface polymerization rate and residual amount of polymer on the plasma-treated surface. Similar results were obtained in Ref. [16] by experiments. In contrast to the above case, an increase in $y_{Ar}$ in CHF_3_ + Ar and C_4_F_8_ + Ar plasmas leads to a nearly proportional decrease in the parameter $\Gamma_{pol}/\sqrt{M_{i}\varepsilon_{i}}\Gamma_{+}\Gamma_{F}$ (by ~3.3 times and ~3.8 times, respectively, at 0–75% Ar) and, thus, causes a similar change in $h_{pol}$. As such, the variation of $y_{Ar}$ in these gas systems provides a real tool to adjust both polymer film thickness and $h_{pol}$-sensitive output parameters of the RIE process. It is also worth mentioning that our model-based conclusion on the higher polymerizing ability of C_4_F_8_ + Ar plasma compared with CHF_3_ + Ar one has the direct experimental confirmation. In particular, Ref. [16] reported that the treatment in CHF_3_ plasma causes 1.5–2 times lower $h_{pol}$ values on Si, SiO_2_ and Si_3_N_4_ surfaces compared with those after C_4_F_8_ plasma. The almost identical value of ~2.1 follows from the ratio of $\Gamma_{pol}/\sqrt{M_{i}\varepsilon_{i}}\Gamma_{+}\Gamma_{F}$ parameters for corresponding gas systems, as can be seen from Figure 3d. Therefore, the good correlation between the model-predicted and experimental data allows one to assume that the gas-phase-related parameters shown in Figure 3 provide adequate tracing of the polymer deposition and decomposition kinetics in given gas systems.

**Figure 4:**
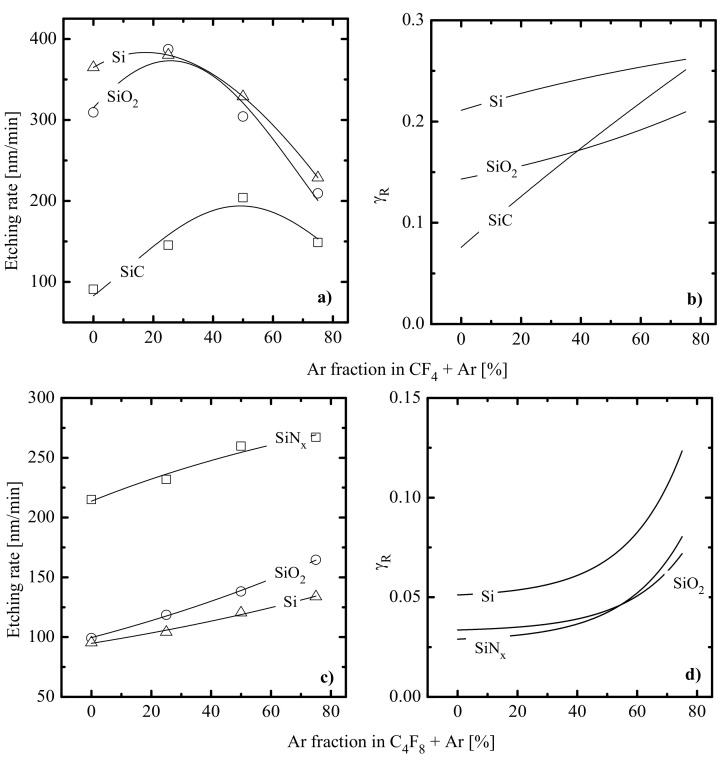
Etching rates (**a**,**c**) and effective reaction probabilities for F atoms (**b**,**d**) for various Si-based materials in CF_4_ + Ar and C_4_F_8_ + Ar plasmas. In (**a**,**b**): CF_4_ + Ar plasma at p = 6 mTorr, W = 700 W and $W_{dc}$ = 300 W (for SiC and SiO_2_) or p = 10 mTorr, W = 800 W and $W_{dc}$ = 300 W (for Si). In (**b**,**c**): C_4_F_8_ + Ar plasma at p = 6 mTorr, W = 900 W and $W_{dc}$ = 200 W.

Figure 4a–d show etching characteristics for various silicon-based materials in CF_4_ + Ar and C_4_F_8_ + Ar plasmas. Since these ones represent gas systems with the maximum difference in polymerizing ability, corresponding data may serve for better understanding the features of low- and high-polymerizing plasmas. Analysis of Figure 4a,c in light of both previous etching experiences [39,41] and our data concerning fluxes of plasma active species allows one to formulate several principal suggestions about etching mechanisms and related effects. These are as follows:

- Experiments with pure Ar plasma indicated that etching rates for all four materials (in fact, rates of physical sputtering, $R_{phys}$) are quite close and do not exceed 20 nm/min. The correction of corresponding values according to the change of $\sqrt{M_{i}\varepsilon_{i}}\Gamma_{+}$ in fluorocarbon-containing plasmas (see Figure 3c) confirmed that the range of 0–75% Ar is characterized by $R_{phys}$ << $R_{chem}$ and $R\approx R_{chem}$. As such, the dominant etching pathway in all cases is the ion-assisted chemical reaction.
- Quite similar $R_{chem}=f\left(y_{Ar}\right)$ curves within the given gas system may be caused by the fact that corresponding etching processes are driven by identical active species and have the same limiting stage. A similar conclusion was made in the experimental study of Standaert et al. [16] for Si, SiO_2_ and Si_3_N_4_ in both CHF_3_ and C_4_F_8_ plasmas. At the same time, the evident dissimilarity in etching rate behaviors in Figure 4a,c points to different etching mechanisms in CF_4_- and C_4_F_8_-based plasmas. This may be, for example, the ion-assisted chemical reaction under the condition of thin or even non-continuous polymer film (in the case of low-polymerizing CF_4_ + Ar plasma) or the etching regime controlled by the polymer thickness through the transport of etchant species to the film/etched surface interface (in the case of high-polymerizing C_4_F_8_ + Ar plasma).

The non-monotonic Si, SiO_2_ and SiC etching rates from Figure 4a contradict with the monotonically decreasing F atom flux and correspond to monotonically increasing effective reaction probabilities $\gamma_{R}=R_{chem}/\Gamma_{F}$ (Figure 4b). Obviously, the growth of $\gamma_{R}$ in the CF_4_ + Ar plasma cannot be associated with the change in $h_{pol}$ because of nearly constant values for both $\Gamma_{pol}/\Gamma_{F}$ (Figure 3b) and $\Gamma_{pol}/\sqrt{M_{i}\varepsilon_{i}}\Gamma_{+}\Gamma_{F}$ (Figure 3d) in the range of 0–75% Ar. At the same time, $\gamma_{R}$ shows an evident agreement with the behavior of $\sqrt{M_{i}\varepsilon_{i}}\Gamma_{+}$ (Figure 3c) that reflects an increase in the ion bombardment intensity toward Ar-rich plasmas. All these allow one to conclude that the F + Si/SiO_2_/SiC reaction kinetics are not influenced by surface polymerization but are sensitive to ion bombardment through desorption of reaction products and the formation of adsorption sites for F atoms. In fact, such a situation represents a typical case of ion-assisted chemical reaction in non-polymerizing gas systems [9,69,70]. One should also mention that similar etching behaviors for Si and SiO_2_ surely mean that the production of adsorption sites for F atoms in the ion-assisted process SiO_x_(s.) → Si(s.) + xO does not limit the SiO_2_ etching rate. In our opinion, such a situation is due to high ion bombardment intensity.

The monotonically increasing Si, SiO_2_ and SiN_x_ etching rates from Figure 4c also contradict the change in $\Gamma_{F}$ and also correspond to increasing effective reaction probabilities for all three materials (Figure 4d). The main difference with a previous case is that the change in $\gamma_{R}$ not only follows the tendency of $\sqrt{\varepsilon_{i}}\Gamma_{+}$ but also demonstrates an agreement with a decrease in $h_{pol}$, as can be seen from Figure 3d. Taking into account the high polymerizing ability of C_4_F_8_-based plasmas, one can reasonably suggest that (a) the RIE process in pure C_4_F_8_ plasma is limited by the transport of etchant species through the thick polymer film and (b) the transition to Ar-rich plasmas activates a chemical reaction by providing better access of F atoms to the etched surface. As such, the formally similar changes in $\gamma_{R}$ vs. $y_{Ar}$ in CF_4_ + Ar and C_4_F_8_ + Ar plasmas are caused by different mechanisms.

**Figure 5:**
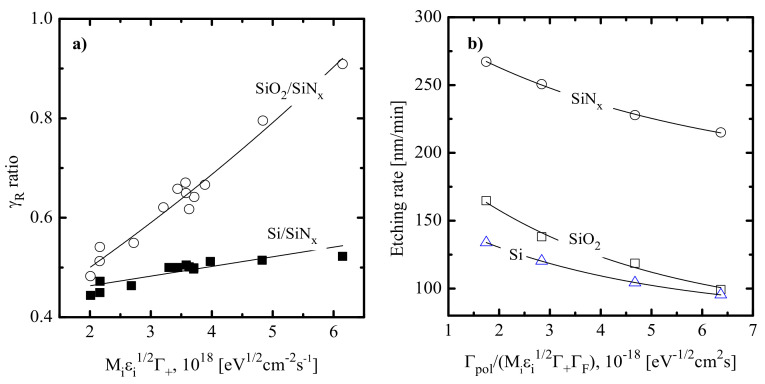
Correlations between gas-phase parameters and heterogeneous process characteristics for SiN_x_, SiO_2_ and Si in C_4_F_8_ + Ar plasma. In (**a**): effective reaction probability ratios vs. parameter $\sqrt{\varepsilon_{i}}\Gamma_{+}$ which traces the intensity of ion bombardment. In (**b**): etching rates vs. parameter $\Gamma_{pol}/\sqrt{\varepsilon_{i}}\Gamma_{+}\Gamma_{F}$ which traces the thickness of polymer film.

In order to confirm the above assumption on etching mechanisms in the C_4_F_8_ + Ar plasma, one can simply correlate our experimental data with those from published works [15,16,17,18,19,20,21,22,23]. In fact, an assumption of $R\approx \gamma_{R}\Gamma_{F}$ implies that ratios of measured etching rates for SiN_x_, SiO_2_ and Si are nearly equal to ratios of corresponding reaction probabilities. In particular, Figure 5a demonstrates the sensitivity of Si/SiN_x_ and SiO_2_/SiN_x_ etching rate ratios (in fact, the sensitivity of $\gamma_{R,Si}/\gamma_{R,SiN_{x}}$ and $\gamma_{R,SiO_{2}}/\gamma_{R,SiN_{x}}$) to the parameter $\sqrt{\varepsilon_{i}}\Gamma_{+}$ characterizing the ion bombardment intensity. Even without looking at these data, one can reasonably expect that stronger sensitivity indicates a bigger difference in both etching regimes and process limiting stages for the given couple of materials. As a confirmation of this suggestion, one can see that an increase in the ion bombardment intensity just slightly increases the $\gamma_{R,Si}/\gamma_{R,SiN_{x}}$ ratio in the range of 0.44–0.52, or by ~1.2 times. As the interaction of F atoms with both Si and SiNx surfaces has a nearly spontaneous (without the energy threshold) nature, the above effect may be attributed to the slower growth in $\gamma_{R,SiN_{x}}$ compared with $\gamma_{R,Si}$ because $h_{pol,SiN_{x}}$ < $h_{pol,Si}$ [16,20]. In our opinion, it looks quite reasonable that the ion bombardment produces a weaker effect in the case of thinner polymer films, since the latter creates fewer problems for the penetration of etchant species to the treated surface. The change in the $\gamma_{R,SiO_{2}}/\gamma_{R,SiN_{x}}$ ratio (0.48–0.95, or by ~2 times) exhibits the formal contradiction with $h_{pol,SiO_{2}}$ < $h_{pol,SiN_{x}}$ [16,20] but also has a reasonable explanation. In fact, since the direct interaction between SiO_2_ and F atoms is thermodynamically forbidden, the corresponding chemical etching mechanism is provided by the breaking of oxide bonds by ion bombardment [9,11,12,13]. That is why the much faster increase in $\gamma_{R,SiO_{2}}$ compared with $\gamma_{R,SiN_{x}}$ with the change in $\sqrt{\varepsilon_{i}}\Gamma_{+}$ may be related to the acceleration of the ion-stimulated process SiO_x_(s.) → Si(s.) + xO and the increasing amount of adsorption sites for F atoms. Obviously, the latter increases the probability of chemical reaction Si(s.) + xF → SiF_x_. In addition, Figure 5b represents all three etching rates as functions of the $\Gamma_{pol}/\sqrt{\varepsilon_{i}}\Gamma_{+}\Gamma_{F}$ ratio, which traces the steady-state thickness of polymer film. The shapes of these curves as well as the decreasing tendencies themselves (that means the thicker polymer film, the lower the etching rate) are in good agreement with those obtained by experiments in Refs. [15,16,20,71]. All these reveal that (a) chemical etching pathways for SiN_x_, SiO_2_ and Si in C_4_F_8_ + Ar plasma are influenced by the polymer film thickness and are characterized by the $h_{pol}$-dependent reaction probability; and (b) the gas-phase-related parameter $\Gamma_{pol}/\sqrt{\varepsilon_{i}}\Gamma_{+}\Gamma_{F}$ is an appropriate pointer to trace the steady-state $h_{pol}$ value.

**Figure 6:**
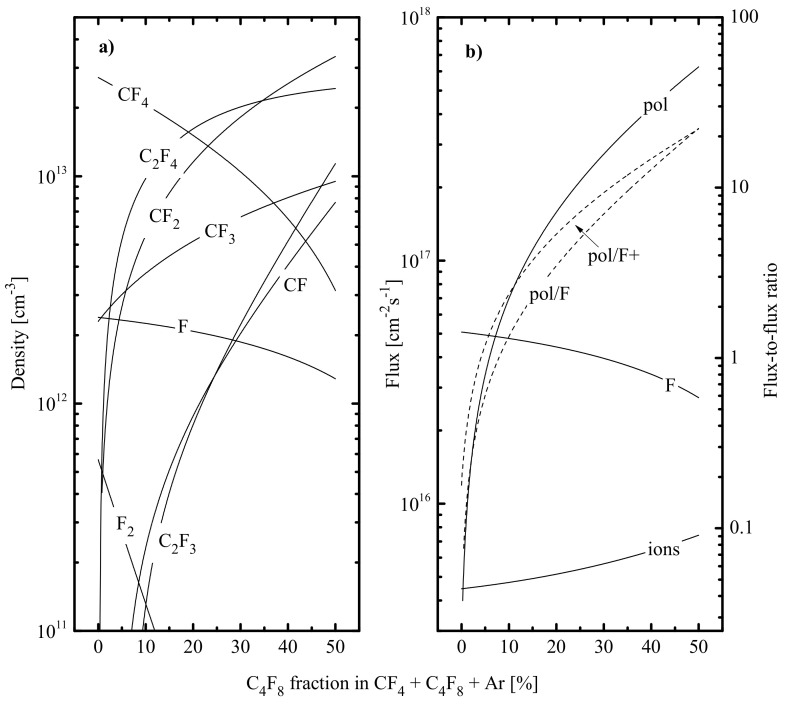
Densities (**a**), fluxes (b, solid lines) and flux-to-flux ratios (b, dashed lines) of active species in CF_4_ + C_4_F_8_ + Ar plasma at p = 4 mTorr, W = 800 W, $W_{dc}$ = 150 W and $y_{Ar}$ = 50%. In (**b**): “pol”—polymerizing radicals; “F”—fluorine atoms; “ions”—positive ions; “pol/F”—$\Gamma_{pol}/\Gamma_{F}$ ratio characterizing polymer deposition rate; “pol/F+”—parameter $\Gamma_{pol}/\sqrt{\varepsilon_{i}}\Gamma_{+}\Gamma_{F}$ (×10^−17^) characterizing the change in polymer film thickness due to the physical etching pathway under the condition of $M_{i}$ ≈ const.

Since effective probabilities of ion-assisted chemical reactions in CF_4_ + Ar and C_4_F_8_ + Ar plasmas are influenced by different factors, one can suggest that the CF_4_/C_4_F_8_ mixing ratio in the three-component CF_4_ + C_4_F_8_ + Ar gas system may also be used to adjust the output characteristics of the RIE process. From Table 2, it can be seen that an increase in $y_{C_{4}F_{8}}$ in the range of 0–50% (in fact, the full substitution of CF_4_ for C_4_F_8_) leads to a noticeable increase in both electron temperature and ion current density. An increase in $T_{e}$ toward C_4_F_8_-rich plasmas may result from decreasing electron energy losses for vibrational and electronic excitation of dominant neutral species, as the gas phase is enriched by less saturated radicals (Figure 6a). The growth of $J_{+}$ follows the behavior of positive ion density due to the same change in the total ionization rate. The last phenomenon is due to (a) differences in ionization rate coefficients for dominant neutral components ($k_{2}$ < $k_{3}$ and $k_{21}$, where R21: C_2_F_4_ + e → C_2_F_3_^+^ + F + 2e) and (b) an increase in ionization rate coefficients for all neutral components together with $T_{e}$. As such, the simultaneous increase in both ion flux and ion bombardment energy (that follows from the change in negative dc bias voltage at $W_{dc}$ = const.) provides sufficient (by ~2 times for 0–50% C_4_F_8_) intensification of ion bombardment.

The data of Figure 6a indicate that an increase in $y_{C_{4}F_{8}}$ lowers the density of CF_4_ molecules as well as leads to the rapid growth of $n_{CF_{2}}$ (3.4 × 10^11^ cm^−3^–3.3 × 10^13^ cm^−3^, or by ~100 times at 0–50% C_4_F_8_) and $n_{C_{2}F_{4}}$ (6.9 × 10^10^ cm^−3^–2.3 × 10^13^ cm^−3^, or by ~330 times 0–50% C_4_F_8_). The reason is that both species directly appear from C_4_F_8_ in R15 and R16, while CF_2_ is also the main dissociation product of C_2_F_4_ in R18. The decreasing dissociation rate of CF_4_ molecules (3.4 × 10^14^ cm^−3^·s^−1^ –1.8 × 10^14^ cm^−3^·s^−1^ totally in R6 and R7 at 0–50% C_4_F_8_) suppresses the formation of CF_3_ radicals but does not result in a decrease in $n_{CF_{3}}$ (2.4 × 10^12^ cm^−3^–9.2 × 10^12^ cm^−3^, or by ~4 times at 0–50% C_4_F_8_. The reason is the increasing rate of R9 that rapidly transforms CF_2_ into CF_3_. The increasing rate of R6 for x = 2 also provides an increase in $n_{CF}$ that contributes to the total density of polymerizing radicals at $y_{C_{4}F_{8}}$ > 25–30%). Accordingly, the growth of $n_{CF}+\;n_{CF_{2}}$ results in the same change for the total flux of polymerizing radicals, as shown in Figure 6a. As for the kinetics of fluorine atoms, it also looks quite understandable in light of the above data on CF_4_ + Ar and C_4_F_8_ + Ar plasmas. In particular, an increase in electron density toward C_4_F_8_-rich plasmas as well as higher $k_{6}$ values for less saturated CF_x_ species provide a change in the dominant formation mechanism for F atoms (from R6 for x = 4 and R7 to R6 for x = 2, 3 at $y_{C_{4}F_{8}}$ > 20%) and an increase in the total formation rate for these species (4.5 × 10^14^ cm^−3^·s^−1^–3.0 × 10^15^ cm^−3^·s^−1^, or by ~7 times 0–50% C_4_F_8_). The opposite change in F atoms’ density (by ~2 times 0–50% C_4_F_8_) is due to the rapid increase in their decay frequency in both heterogeneous (R9) and gas-phase (R20 and R22: C_2_F_3_ + F → C_2_F_4_) processes. This fact is in good agreement with experimental results for CF_4_ + Ar and C_4_F_8_ + Ar plasmas at input powers below 1000 W [68]. Accordingly, the same decreasing trend also occurs for the F atom flux (Figure 5b). It can also be seen that an increase in $y_{C_{4}F_{8}}$ is accompanied by a rapid increase in the polymer deposition rate (as follows from the change of $\Gamma_{pol}/\Gamma_{F}$ ratio) and $h_{pol}$ (as follows from the change of the parameter $\Gamma_{pol}/\sqrt{\varepsilon_{i}}\Gamma_{+}\Gamma_{F}$). All of these surely point to the transition from low- to high-polymerizing plasma.

**Figure 7:**
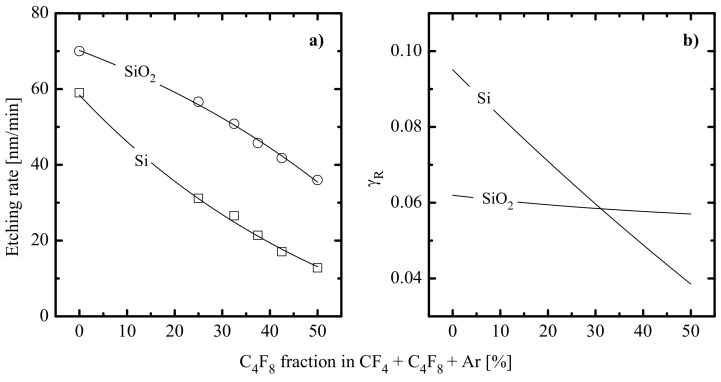
Etching rates (**a**) and effective reaction probability of F atoms (**b**) for Si and SiO_2_ in CF_4_ + C_4_F_8_ + 50% Ar plasma at p = 4 mTorr, W = 800 W and $W_{dc}$ = 150 W.

From etching experiments (Figure 7a), it was found that an increase in $y_{C_{4}F_{8}}$ causes a monotonic decrease in both Si and SiO_2_ etching rates (59–13 and 70–36 nm/min, respectively, at 0–50% C_4_F_8_) as well as a more than twofold increase in SiO_2_/Si etching selectivity (1.2–2.8 at 0–50% C_4_F_8_). Considering that corresponding etching processes are provided by the ion-assisted chemical reaction of F atoms, the obtained differences in etching kinetics may be related to different behaviors of effective reaction probabilities. In fact, the data of Figure 7b indicate only a weak decrease in $\gamma_{R,SiO_{2}}$ (0.061–0.057 at 0–50% C_4_F_8_) as well as the rapid fall of $\gamma_{R,Si}$ (0.0981–0.038 at 0–50% C_4_F_8_). Obviously, the behavior of $\gamma_{R,Si}$ correlates with increasing polymer film thickness. In other words, the substitution of CF_4_ for C_4_F_8_ changes the limiting stage for the Si etching process from the chemical reaction itself (in the case of low $h_{pol,\;Si}$ in 50% CF_4_ + 50% Ar plasma) to the transport of F atoms through the thick polymer film. Accordingly, the continuously increasing $h_{pol,\;Si}$ toward C_4_F_8_- rich plasmas lowers $\gamma_{R,Si}$ through decreasing $\Gamma_{F}{}^{\prime }/\Gamma_{F}$ ratio, where $\Gamma_{F}{}^{\prime }$ is the flux of F atoms on the polymer film/etched surface interface. As a result, the Si etching rate decreases much faster compared with the F atom flux coming from a gas phase. At the same time, the condition $h_{pol,SiO_{2}}$ << $h_{pol,Si}$ sufficiently weakens corresponding effects in the case of SiO_2_. As such, one can obtain a nearly constant $\gamma_{R,SiO_{2}}$ (probably due to $\Gamma_{F}{}^{\prime }/\Gamma_{F}$ ≈ const.) as well as a good quantitative correlation between corresponding etching rate and $\Gamma_{F}.$ Therefore, the change in the CF_4_/C_4_F_8_ mixing ratio in the CF_4_ + C_4_F_8_ + Ar gas system increases the differences between Si and SiO_2_ etching mechanisms as well as providing an effective tool to adjust the SiO_2_/Si etching selectivity.

### 3.2. Oxygenated Gas Systems

The subjects of this chapter are the ternary CF_4_ + O_2_ + Ar, CHF_3_ + O_2_ + Ar and C_4_F_8_ + O_2_ + Ar gas mixtures with a constant 50% fraction of the fluorocarbon component. Accordingly, the main variable parameter in all three cases was the O_2_/Ar mixing ratio in order to illustrate how the substitution of inert gas for the chemical active one influences gas-phase plasma parameters and RIE process characteristics. It is important to note that such a gas mixing regime is different compared with that studied for many binary fluorocarbon + O_2_ gas systems where an increase in $y_{O_{2}}$ corresponds to a proportional change in the fraction of the fluorocarbon component.

**Figure 8:**
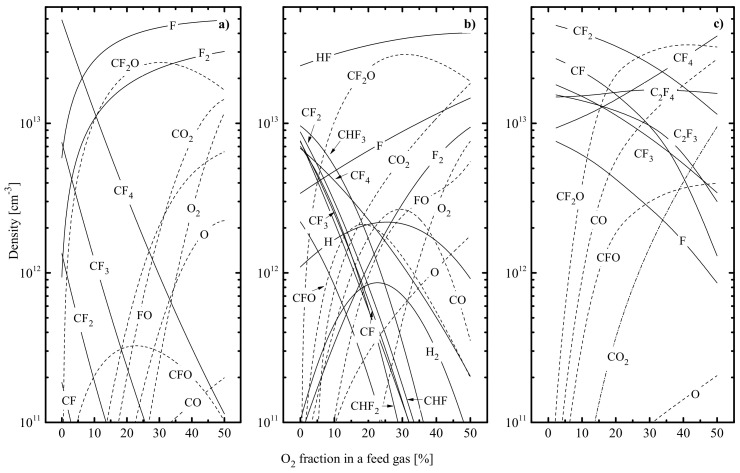
Densities of neutral species in 50% CF_4_ + O_2_ + Ar (**a**), 50% CHF_3_ + O_2_ + Ar (**b**) and 50% C_4_F_8_ + O_2_ + Ar (**c**) plasmas at p = 6 mTorr, W = 700 W and $W_{dc}$ = 200 W. The change in $y_{O_{2}}$ in the range of 0–50% corresponds to the full substitution of Ar for O_2_.

**Figure 9:**
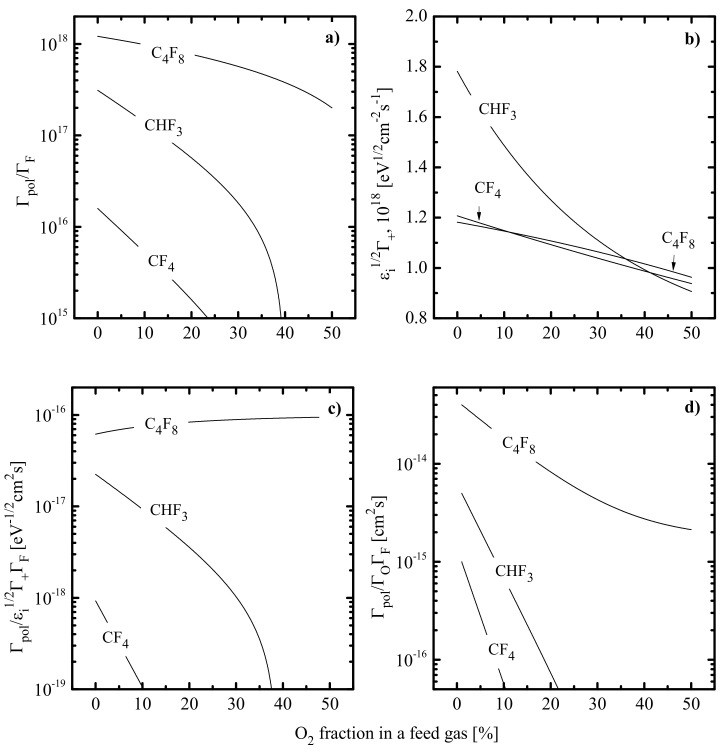
Fluxes and flux-to-flux ratios characterizing etching and polymerization kinetics in 50% CF_4_ + O_2_ + Ar (**a**), 50% CHF_3_ + O_2_ + Ar (**b**) and 50% C_4_F_8_ + O_2_ + Ar (**c**) plasmas at p = 6 mTorr, W = 700 W and $W_{dc}$ = 200 W: (**a**) $\Gamma_{pol}/\Gamma_{F}$ ratio characterizing polymer deposition rate; (**b**) parameter $\sqrt{\varepsilon_{i}}\Gamma_{+}$ characterizing polymer etching rate under the condition of $M_{i}$ ≈ const.; (**c**) parameter $\Gamma_{pol}/\sqrt{\varepsilon_{i}}\Gamma_{+}\Gamma_{F}$ characterizing the change in polymer film thickness due to the physical etching pathway; (**d**) parameter $\Gamma_{pol}/\Gamma_{O}\Gamma_{F}$ characterizing the change in polymer film thickness due to the chemical etching pathway.

From Table 3, it can be understood that the substitution of Ar for O_2_ in all three gas mixtures results in similar changes in electron- and ion-related plasma parameters. Corresponding results may be briefly commented as follows:

- A decrease in $T_{e}$ toward O_2_-rich plasmas results from an increase in electron energy losses in low-threshold inelastic processes, such as vibrational and electronic excitations of both O_2_ itself and molecular products of plasma chemical reactions—CO, CO_2_, FO and CF_x_O (Figure 8). In particular, the lowest excitation potential for Ar is ~11.6 eV, while O_2_ is characterized by the almost continuous energy loss spectrum from ~0.2 eV. Such a situation is due to the vibration excitation R23: O_2_ (V = 0) + e → O_2_ (V > 0) + e ($\varepsilon_{23}$ = 0.16 eV) as well as by the formation of metastable states in R24: O_2_ + e → O_2_ (a^1^∆) + e ($\varepsilon_{24}$ = 0.98 eV) and R25: O_2_ + e → O_2_(b^1^∑) + e ($\varepsilon_{25}$ = 1.64 eV).
- A decrease in plasma density toward higher $y_{O_{2}}$ values is provided by a combination of two evident mechanisms. First, the decreasing $T_{e}$ lowers the overall ionization efficiency through the change in ionization rate coefficients, $k_{iz}$, for all neutral gas-phase components. The high sensitivity of $k_{iz}$, to $T_{e}$ is because typical threshold energies ($\varepsilon_{iz}\;$≈ 12–15 eV [9]) exceed the mean electron energy (3/2$)T_{e}$. Secondly, an increase in $y_{O_{2}}$ introduces many electronegative species in the form of O- and F-containing reaction products. This probably accelerates losses of both electrons and positive ions in the dissociative attachment and ion–ion recombination processes. Ion current densities and ion fluxes in all three gas systems also exhibit decreasing tendencies, according to the behavior of $n_{+}$.
- An increase in negative dc bias is mainly due to the opposite change in ion flux. The reason is that the lower ion flux leads to weaker compensation for the excess negative charge provided by bias power source at $W_{dc}$ = const. The corresponding increase in ion bombardment energy does not overlap with the change in $\Gamma_{+}$, so the parameter $\sqrt{\varepsilon_{i}}\Gamma_{+}$ characterizing the intensity of ion bombardment at $M_{i}$ ≈ const. also shows a decrease toward O_2_-rich plasmas (Figure 9).

Figure 8 illustrates the effect of $y_{O_{2}}$ on steady-state densities of neutral species. Common features of all three gas systems are that the transition toward O_2_-rich plasmas (a) suppresses densities of fluorocarbon radicals and (b) provides the formation of CO_2_, CO, FO and CF_x_O molecules, with CF_2_O as a dominant component. At the same time, the kinetics of F atoms exhibit principal differences and are worth brief discussion.

In the CF_4_ + O_2_ + Ar plasma, the substitution of Ar for O_2_ noticeably reduces the F atom formation rate in R6 and R7 even under the condition of $y_{O_{2}}$ < $y_{Ar}$. This is due to the simultaneous decrease in $n_{e}$, $n_{CF_{4}}$ and $n_{CF_{3}}$ (Figure 8a). The decreasing density of CF_3_ radicals is the result of their conversion into CF_x_O species through R26: CF_x_ + O → CF_x−1_O + F, R27: CF_x_ + O(^1^D) → CF_x−1_O + F, R28: CF_3_ + CFO → CF_4_ + CO and R29: CF_3_ + CFO → CF_2_O + CF_2_ with the participation of O, O(^1^D), and CFO itself. The change in $n_{CF_{4}}$ is reasonably similar with that for $n_{CF_{3}}$, as the latter provides the formation of CF_4_ molecules in both gas-phase and heterogeneous recombination pathways. In addition to R26 and R27, an increase in $y_{O_{2}}$ sufficiently affects F atoms’ kinetics by (a) introducing new formation channels in the form of electron impact processes R30: CFO + e → CO + F + e and R31: CF_2_O + e → CFO + F + e and (b) increasing efficiency of R32: F_2_ + e → 2F + e. High formation rates and densities for CFO species are provided by R31 and R33: CO + F → CFO, while the same effect for CF_2_O molecules results from R29, R34: 2CFO → CF_2_O + CO and R35: CFO + F → CF_2_O. The acceleration of R32 results from the rapidly increasing F_2_ density due to R36: CF_2_O + O(^1^D) → F_2_ + CO_2_ and heterogeneous recombination of F atoms. The transition to highly oxygenated plasmas ($y_{O_{2}}$ > $y_{Ar}$) maintains all the previously mentioned tendencies for reaction rates as well as adding one more reaction pathway contributing to the F atom formation rate, such as R37: FO + e → F + O + e. The high formation rate and density of FO species in O_2_-rich plasmas are provided mainly by R38: F_2_ + O(^1^D) → FO + F and the heterogeneous interaction between F and O atoms. As a result, the total effect from R30, R31 and R37 becomes greater than the sum of R6 and R7. Apart from these, the rates of the atom molecular processes R38, R39: FO + O → F + O_2_, R40: FO + O(^1^D) → F + O_2_, R41: 2FO → 2F + O_2_ and R42: CFO + O → CO_2_ + F increase together with $y_{O_{2}}$ and, finally, reach levels of R30, R31 and R37. As a result, the substitution of Ar for O_2_ leads to the monotonic increase in the F atom production rate as well as causing the same change in the F atom density (Figure 8a). It is important to note, also, that the opposite behaviors of $n_{F}$ and $n_{CF}+n_{CF_{2}}$ surely lower the polymer deposition rate (as can be seen from Figure 9a which indicates a decrease in the $\Gamma_{pol}/\Gamma_{F}$ ratio) and create a favorable condition for the residual-less etching.

In the CHF_3_ + O_2_ + Ar plasma, an increase in O_2_ content in feed gas also retards the electron impact dissociation kinetics (to a greater extent than in previous cases because of deeper falls in both $T_{e}$ and $n_{e}$) and rapidly reduces the densities of CF_x_ and CHF_x_ species (Figure 8b). The fast loss of CHF_x_ radicals is due to their effective conversion into CF_x_O species through R43: CHF_x_ + O → CF_x_O + H and R44: CHF_x_ + O → CF_x−1_O + HF. The same effect for CF_x_ (for example, 6.3 × 10^12^ cm^−3^–7.0 × 10^9^ cm^−3^, or by ~900 times for CF_3_ and 7.3 × 10^12^ cm^−3^–5.3 × 10^9^ cm^−3^, or by ~1400 times at 0–50% O_2_) is connected with increasing their decay rates due to R26 and R27 as well as decreasing formation rates in CHF_x_ + F → CF_x_ + HF (R11). Accordingly, the total effect of CF_x_ + e → CF_x−1_ + F + e (R6) and R45: CHF_x_ + e → CHF_x−1_ + F + e in respect to the production of F atoms shows a decrease with increasing $y_{O_{2}}$ value. In addition, though the increasing rate of R44 provides weak growth in the density of HF molecules, the F atom formation rate through HF + e → H + F + e (R14) also goes down due to the tenfold fall in $k_{14}n_{e}$ (88.5–8.3 s-1 at 0–50% O_2_). Therefore, the substitution of Ar for O_2_ strongly suppresses all F atom formation pathways which work in the CHF_3_ + Ar plasma. Another remarkable fact is the much lower efficiency of additive F atom formation pathways with a participation of oxygen-containing species. In particular, the non-principal contributions of R31 and R23 are because corresponding rates are limited by the formation of CF_2_O species in R35 and the corresponding heterogeneous counterpart. Their total rate reaches the maximum at 20–30% O_2_ and remains lower compared with that for R14. Similarly, contributions of R37 and R39–R41 are limited by the formation rate of FO molecules in heterogeneous recombination of atomic species. The latter is also less effective compared with previous gas systems due to (a) lower O and O(^1^D) production rates in R46: O_2_ + e → 2O + e and R47: O_2_ + e → O + O(^1^D) + e and b) faster decay of oxygen atoms in R26, R27, R43 and R44. All of these result in a decrease in the total F atom formation rate toward O_2_-rich plasmas (4.5 × 10^15^ cm^−3^·s^−1^–2.0 × 10^15^ cm^−3^·s^−1^, or by ~2.3 times at 0–50% O_2_). At the same time, rapidly decreasing densities of CHF_x_ and CF_x_ radicals reduce the effective decay frequency for F atoms in both heterogeneous (R9) and gas-phase (R11) reactions. Since the last tendency appears to be faster compared with a decrease in the F atom formation rate, a monotonic increase in both F atom density (Figure 8b) and flux takes place. Opposite tendencies for ${\;\Gamma }_{pol}$ and $\Gamma_{F}$ provide a rapid decrease in the ${\;\Gamma }_{pol}/\Gamma_{F}$ ratio toward O_2_-rich plasmas (Figure 9a). This corresponds to decreasing polymer deposition rate and plasma polymerizing ability.

In the C_4_F_8_ + O_2_ + Ar plasma, an increase in $y_{O_{2}}$ noticeably reduces rates of R6 (due to the simultaneous decrease in $T_{e}$ and $n_{e}$) as well as introducing additional pathways for the decomposition of CF_x_ radicals through CF_x_ + O → CF_x−1_O + F (R26) and CF_x_ + O(^1^D) → CF_x−1_O + F (R27). This provides the monotonic decrease in $n_{CF_{x}}$ (4.7 × 10^13^ cm^−3^–1.2 × 10^13^ cm^−3^ for x = 2 and 2.9 × 10^13^ cm^−3^–1.3 × 10^12^ cm^−3^ for x = 1 at 0–50% O_2_, see Figure 8c) which, however, appears to be slower compared with CF_4_ + O_2_ + Ar and CHF_3_ + O_2_ + Ar plasmas. The reason is that the effective loss of O_2_ molecules through R48: CF + O_2_ → CFO + O ($k_{48}$ ~ 3.2 × 10^−11^ cm^3^/s) and R49: C + O_2_ → CO + O ($k_{49}$ ~ 2.0 × 10^−11^ cm^3^/s) limits the formation rates for O and O(^1^D) species in R46 and R47. The luck of oxygen atoms reduces the effect of R26, R27, R30 and R31 on the F atom formation kinetics (in fact, it provides the condition $R_{6}$ > $R_{26}$ + $R_{27}$ + $R_{30}$ + $R_{31}$ for 0–50% O_2_) and, thus, leads to monotonically decreasing F atom density and flux. Though changes in ${\;\Gamma }_{pol}$ and ${\;\Gamma }_{F}$ are qualitatively similar, the latter appears to be slower on a quantitative scale. Such a situation corresponds to the weakly decreasing ${\;\Gamma }_{pol}/\Gamma_{F}$ ratio (Figure 8a) that points to the same behavior of polymer deposition rate.

The data of Figure 9 allow one to summarize features of polymer deposition and decomposition kinetics in given gas systems. First, CF_4_-, CHF_3_- and C_4_F_8_- based plasmas in the presence of oxygen exhibit the same order of their polymerizing abilities, as was mentioned for non-oxygenated binary mixtures with Ar. As such, the addition of O_2_ up to 50% does not change the basic rule concerning the correlation between the polymerizing ability and the z/x ratio in original C_x_H_y_F_z_ molecules. Second, the substitution of Ar for O_2_ in all three gas systems suppresses the polymer deposition rate through increasing the gap between densities of F atoms and polymerizing radicals. The stronger effect for CF_4_ + O_2_ + Ar and CHF_3_ + O_2_ + Ar plasmas is because of the increasing density of F atoms and opposite changes in ${\;\Gamma }_{pol}$ and ${\;\Gamma }_{F}$ toward higher $y_{O_{2}}$ values. Thirdly, the substitution of Ar for O_2_ in all three gas systems results in decreasing polymer film thickness. Again, the stronger change in $h_{pol}$ for CF_4_ + O_2_ + Ar and CHF_3_ + O_2_ + Ar plasmas is due to the simultaneous acceleration in physical (Figure 9c) and chemical (Figure 9d) polymer decomposition pathways. In the C_4_F_8_ + O_2_ + Ar plasma, the decrease in $\sqrt{\varepsilon_{i}}\Gamma_{+}$ has almost the same slope as the change in ${\;\Gamma }_{pol}/\Gamma_{F}$ ratio. Such a situation causes nearly constant efficiency for sputter etching of polymer film in the range of 0–50% O_2_.

**Figure 10:**
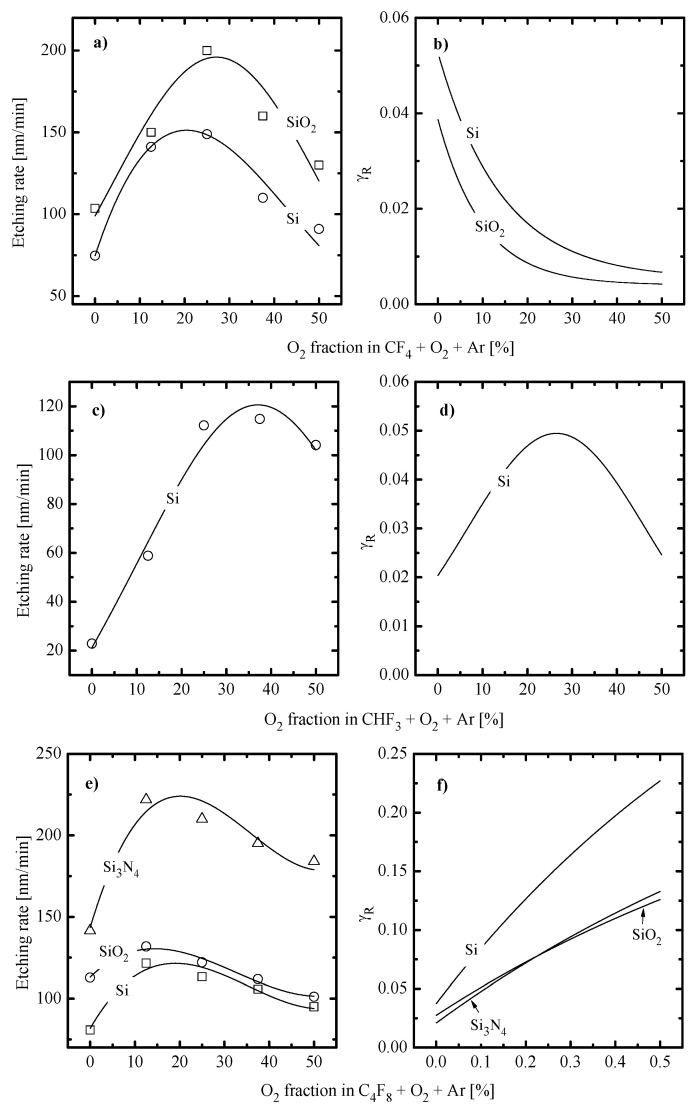
Etching rates (**a**,**c**,**e**) and effective reaction probabilities for F atoms (**b**,**d**,**f**) for various Si-based materials in oxygenated fluorocarbon plasmas. In (**a**,**b**): 50% CF_4_ + O_2_ + Ar plasma at p = 6 mTorr, W = 700 W and $W_{dc}$ = 200 W. In (**b**,**c**): 50% CHF_3_ + O_2_ + Ar plasma at p = 6 mTorr, W = 700 W and $W_{dc}$ = 200 W. In (**e**,**f**): 50% C_4_F_8_ + O_2_ + Ar plasma at p = 10 mTorr, W = 700 W and $W_{dc}$ = 200 W.

From Figure 10a,c,e, it can be seen that an increase in $y_{O_{2}}$ results in non-monotonic etching rates of Si, SiO_2_ and Si_3_N_4_ for all three gas mixtures. Taking into account that the rule $R_{phys}$ << $R_{chem}$ also works here, one can surely speak about the non-constant $\gamma_{R}$ that depends on the feed gas composition. In the case of low-polymerizing CF_4_ + O_2_ + Ar plasma, the decreasing $\gamma_{R}$ for Si and SiO_2_ (Figure 10b) contradicts with the change in $h_{pol}$ (since the latter also decreases toward O_2_-rich plasmas) but shows formal agreement with $\sqrt{\varepsilon_{i}}\Gamma_{+}$. At the same time, one can suggest that quite similar $\gamma_{R}=f\left(y_{O_{2}}\right)$ curves for Si and SiO_2_ mean identical mechanisms are influencing the behaviors of effective reaction probabilities for these materials, while spontaneous F + Si reaction kinetics are less sensitive to the ion bombardment intensity.

In our opinion, the similar decrease in both $\gamma_{R,Si}$ and $\gamma_{R,SiO_{2}}$ is only partly supported by the change in ion bombardment intensity, but is mostly caused by heterogeneous effects with a participation of oxygen atoms. When assuming the rather thin or even the non-continuous fluorocarbon polymer film which does not influence the etching kinetics, there are at least two reasonable mechanisms which should be considered. The first one is the competitive adsorption of O atoms with a formation of oxide bonds R50: Si(s.) + O → SiO(s.), while the back process R51: SiO(s.) → Si(s.) + O has an ion-driven nature. Obviously, an increase in $y_{O_{2}}$ accelerates R50 (due to an increase in O atom flux, see Figure 7a) under the condition of a slightly decreasing reaction yield for R51, as follows from the change in $\sqrt{\varepsilon_{i}}\Gamma_{+}$. As a result, the transition toward O_2_-rich plasmas reduces the amount of free adsorption sites for etchant species and suppresses $\gamma_{R}$. The second mechanism suggests that an increase in $y_{O_{2}}$ changes the composition of reaction products toward lower volatile compounds. From Refs. [72,73,74], it can be understood that both SiCl_x_O_y_ and SiBr_x_O_y_ compounds have much lower volatility compared with SiCl_x_ and SiBr_x_. Therefore, if the same rule works for the fluorine-based etching chemistry, the oxidation of reaction products R52: SiF_x_(s.) + yO → SiF_x_O_y_(s.) must assume their ion-stimulated desorption pathway R53: SiF_x_O_y_(s.) → SiF_x_O_y_, which is less effective compared with both spontaneous desorption of SiF_x_ (x = 4) and ion-stimulated desorption of SiF_x_ (x < 4). As such, the acceleration of R52 together with decreasing efficiency of R53 increases the residual amount of reaction products on the etched surface and, thus, reduces $\gamma_{R}$ through a decreasing fraction of free adsorption sites for F atoms.

In the case of high-polymerizing C_4_F_8_ + O_2_ + Ar plasma, an increase in $\gamma_{R}$ for Si, SiO_2_ and Si_3_N_4_ (Figure 10d) contradicts $\sqrt{\varepsilon_{i}}\Gamma_{+}$ but shows an agreement with the change in the polymer film thickness. It is probable that even full substitution of Ar for O_2_ still keeps the thick polymer film and, thus, does not change the process limiting stage compared with the oxygen-less C_4_F_8_ + Ar plasma. Accordingly, decreasing $h_{pol}$ with higher O_2_ fractions in a feed gas accelerates the chemical etching pathway by providing better access of F atoms to the etched surface (in fact, by increasing the $\Gamma_{F}{}^{\prime }/\Gamma_{F}$ ratio).

In the case of CHF_3_ + O_2_ + Ar plasma, one can speak about an intermediate case when an increase in $y_{O_{2}}$ provides the transition from thick to thin polymer film. It can be suggested that under the condition of Ar-rich plasma ($y_{O_{2}}$ < $y_{Ar})$, the polymer film is still thick enough to limit the rate of chemical reaction through the transport of F atoms to the etched surface. That is why the increasing tendency of $\gamma_{R}$ up to 25–30% O_2_ for all three materials correlates with the change in $h_{pol}$. On the contrary, O_2_-rich plasma ($y_{O_{2}}$ > $y_{Ar}$) provides a thin polymer film that satisfies the condition of $\Gamma_{F}{}^{\prime }/\Gamma_{F}$ ≈ 1. As such, a further decrease in the amount of residual polymer does not influence both $\gamma_{R}\;$ and kinetics of the chemical etching pathway. At the same time, such a situation lowers the amount of O atoms consumed for the reaction with the fluorocarbon polymer as well as increasing the O atom flux reaching the etched surface. Probably, this stimulates heterogeneous reaction pathways R50–R53 as well as lowering the effective reaction probability for F atoms through the decreasing fraction of free adsorption sites. That is why the $\gamma_{R,Si}=f\left(y_{O_{2}}\right)$ curve exhibits a non-monotonic shape (Figure 10d), and the bend point at about $y_{O_{2}}$ ≈ $y_{Ar}$ reflects the change in the process limiting stage.

## 4. Conclusions

In this work, we summarized and re-discussed, on a comparative scale, the results of our previous studies related to reactive ion etching kinetics and mechanisms for silicon and silicon-based materials (SiC, SiO_2_, Si_x_N_y_ and SiO_x_N_y_) in multi-component fluorocarbon gas mixtures. The latter were represented either by one fluorocarbon component (CF_4_, C_4_F_8_, CHF_3_) with Ar and O_2_ or by two fluorocarbon components with one additive gas. In contrast to “classical” works [39,41,75,76], we did not use surface diagnostics methods, did not measure the polymer film thickness and did not investigate its chemical structure. Instead, we performed a phenomenological study based on correlations between experimentally obtained etching kinetics and model-predicted gas-phase plasma characteristics (ion energy, ion flux, densities and fluxes of F atoms and polymerizing radicals) providing various heterogeneous effects. In our opinion, such an approach serves for a more accurate interpretation of etching mechanisms (with respect to dominant interaction pathways, limiting stages and etching regimes) and, thus, provides an additional opportunity for etching process optimization.

It was demonstrated that in both non-oxygenated and oxygenated gas systems, plasma polymerization ability (the deposition rate and the steady-state thickness of polymer film) correlates with the x/z ratio in the original C_x_H_y_F_z_ molecule. At the same time, F atom kinetics and ions-related plasma parameters (ion flux and ion bombardment energy) are strongly dependent on individual properties of the corresponding fluorocarbon component. In CF_4_ + Ar, CHF_3_ + Ar and C_4_F_8_ + Ar plasmas, an increase in Ar fraction in the feed gas always (a) results in sufficient changes in electron temperature and plasma density (due to changes in both electron energy loss balance and total ionization rate); (b) increases the intensity of ion bombardment; and (c) causes a lower-than-proportional decrease in F atom density. The last feature is due to either an increase in electron impact dissociation frequencies for CF_x_ species (in the case of CF_4_ + Ar plasma) or a decrease in F atom loss frequencies in gas-phase reactions (in the case of CHF_3_ + Ar and C_4_F_8_ + Ar plasmas). In CF_4_ + O_2_ + Ar, CHF_3_ + O_2_ + Ar and C_4_F_8_ + O_2_ + Ar gas mixtures, the substitution of Ar for O_2_ causes similar changes in electron- and ion-related plasma parameters and suppresses densities of polymerizing radicals but has a different impact on F atom kinetics. As such, even the formally similar changes in F atom density for CF_4_ + O_2_ + Ar and CHF_3_ + O_2_ + Ar plasmas result from different plasma chemical reaction mechanisms.

It was found that the dominant etching mechanism in all cases is represented by the chemical etching pathway (since the rate of physical sputtering is much lower compared with the measured etching rate), while the latter does not follow the behavior of F atom flux. This fact points out the process-condition-dependent effective reaction probability for F atoms. The analysis of correlations between the effective reaction probability and fluxes (or flux-to-flux ratios) of plasma active species allows one to suggest the limiting stage of the etching process. Depending on the gas system, this may be the ion-stimulated desorption of reaction products, the transport of F atoms through thick polymer film or heterogeneous reactions with the participation of oxygen atoms under the condition of thin polymer film. Most of the model-predicted results are in good agreement with published experimental data on etching and polymerization kinetics in various fluorocarbon-based plasmas.

## Figures and Tables

**Table 1:** Electron- and ion-related plasma parameters in CF_4_ + Ar, CHF_3_ + Ar and C_4_F_8_ + Ar plasmas at p = 6 mTorr, W = 700 W and $W_{dc}$ = 200 W

$y_{Ar}\;(\%)$	CF_4_ + Ar			CHF_3_ + Ar			C_4_F_8_ + Ar		
	T_e_ (eV)	n_+_ (cm^−3^)	−U_dc_ (V)	T_e_ (eV)	n_+_ (cm^−3^)	−U_dc_ (V)	T_e_ (eV)	n_+_ (cm^−3^)	−U_dc_ (V)
0	3.6	4.4 × 10^10^	262	5.2	5.1 × 10^10^	230	4.7	3.9 × 10^10^	278
25	3.6	4.5 × 10^10^	249	4.9	5.7 × 10^10^	205	4.5	4.4 × 10^10^	249
75	3.8	5.8 × 10^10^	188	4.7	6.9 × 10^10^	180	3.8	8.3 × 10^10^	198

**Table 2:** Electron- and ion-related plasma parameters in CF_4_ + C_4_F_8_ + Ar plasma at p = 4 mTorr, W = 800 W, $W_{dc}$ = 150 W and $y_{Ar}$ = 50%.

$y_{C_{4}F_{8}}$ (%)	T_e_ (eV)	J_+_ (mA/cm^2^)	n_+_ ≈ n_e_ (cm^−3^)	−U_dc_ (V)	$\varepsilon_{i}$ (eV)	$\sqrt{\varepsilon_{i}}\Gamma_{+}\;({\mathrm{eV}}^{1/2}{\mathrm{cm}}^{-2}s^{-1})$
0	3.5	0.73	2.9 × 10^10^	121	141	5.4 × 10^16^
25	3.9	0.83	3.3 × 10^10^	148	169	6.8 × 10^16^
50	4.3	1.21	4.0 × 10^10^	165	190	1.0 × 10^17^

**Table 3:** Electron- and ion-related plasma parameters in 50% CF_4_ + O_2_ + Ar, 50% CHF_3_ + O_2_ + Ar and 50% C_4_F_8_ + O_2_ + Ar plasmas at p = 6 mTorr, W = 700 W and $W_{dc}$ = 200 W.

$y_{O_{2}}\;(\%)$	CF_4_ + O_2_ + Ar			CHF_3_ + O_2_ + Ar			C_4_F_8_ + O_2_ + Ar		
	T_e_ (eV)	n_+_ (cm^−3^)	−U_dc_ (V)	T_e_ (eV)	n_+_ (cm^−3^)	−U_dc_ (V)	T_e_ (eV)	n_+_ (cm^−3^)	−U_dc_ (V)
0	3.6	4.9 × 10^10^	215	4.8	6.2 × 10^10^	190	4.8	4.4 × 10^10^	212
25	3.5	3.9 × 10^10^	239	3.9	3.9 × 10^10^	234	4.0	4.0 × 10^10^	238
50	3.4	3.2 × 10^10^	250	3.0	3.0 × 10^10^	254	3.1	3.7 × 10^10^	269

## Data Availability

The data presented in this study are available on request from the corresponding author.
